# Future land cover change scenarios in South African grasslands – implications of altered biophysical drivers on land management

**DOI:** 10.1016/j.heliyon.2018.e00693

**Published:** 2018-07-17

**Authors:** Lesley Gibson, Zahn Münch, Anthony Palmer, Sukhmani Mantel

**Affiliations:** a School of Engineering, John Muir Building, The King's Buildings, Edinburgh, EH9 3JL, Scotland, United Kingdom; b Department of Geography and Environmental Studies, Stellenbosch University, Stellenbosch, 7600, South Africa; c Centre for African Conservation Ecology, Nelson Mandela University, PO Box 77000, Port Elizabeth 6019, South Africa; d Institute for Water Research, Rhodes University, Grahamstown, 6140, South Africa

**Keywords:** Environmental science, Geography

## Abstract

Future land cover changes may result in adjustments to biophysical drivers impacting on net ecosystem carbon exchange (NEE), catchment water use through evapotranspiration (ET), and the surface energy balance through a change in albedo. The Land Change Modeller (Idrisi Terrset 18.08) and land cover for 2000 and 2014 are used to create a future scenario of land cover for two catchment with different land management systems in the Eastern Cape Province for the year 2030. In the S50E catchment, a dualistic farming system, the trend shows that grasslands represented 57% of the total catchment area in 2014 decreasing to 52% by 2030 with losses likely to favour a gain in woody plants and cultivated land. In T35B, a commercial system, persistence of grasslands is modelled with approximately 80% coverage in both years, representing a more stable system. Finally, for S50E, NEE and ET will increase under this land cover change scenario leading to increased carbon sequestration but less water availability and corresponding surface temperature increases. This implies that rehabilitation and land management initiatives should be targeted in catchments under a dualistic farming system, rather than those which are predominantly commercial systems.

## 1 Introduction

Land use and land cover change (LULCC) has been suggested to be the most important anthropogenic disturbance to the environment at a local level, causing various microclimatic changes (Mishra and Rai, 2016). Anthropogenic influences on the landscape such as alteration in land use through agriculture, forestry, urbanisation and the introduction of invasive alien plant (IAP) species have a profound effect on the functioning of the landscape and ecosystems. Further, the present land cover may affect the movement of species as well as determining the availability of land for future use (Singh et al., 2014). The modifications generally lead to a degraded environment and thus the importance of maintaining the integrity of ecosystems is fundamental to preserve biodiversity (Singh et al., 2014). LULCC has been related to biodiversity loss and thus recent research has arisen to meet land management needs and to assess the role of LULCC in the functioning of the biosphere, through the development of a range of LULCC models (Pérez-Vega et al., 2012). LULCC modelling entails the simulation or prediction of the behaviour of the environmental and social systems in the study area over a time period in such a way that it relates to the measured land change (Paegelow et al., 2013).

In a water scarce country such as South Africa, climate change adaptation is particularly important for catchment management. A change in catchment land cover will have a direct effect on the hydrological functioning of a catchment and thus predicting land cover change may help to develop resilience to projected climate changes through, for example, evidence-based water licensing (Palmer et al., 2017). The principle drivers of change within the rural areas of southern Africa are linked to five primary drivers, namely commercial afforestation, woody encroachment (both alien and native woody plant invasion), urbanization, increased dryland cultivation and rangeland degradation, and it is now well understood that invasion by alien woody plants is a major driver of grassland transformation and influences the ecosystem services (forage production, water supply, habitat, biodiversity, carbon sequestration and recreation) provided by these rangelands (Münch et al., 2017).

The storage of carbon in the landscape is driven by biophysical parameters associated with each land cover type and thus changes in land cover proportions across a catchment will impact on the net ecosystem carbon exchange (NEE) of the catchment as a whole. Similarly, the ecophysiology of the individual land covers affects the water use of the vegetation within that land cover and changes in land cover proportions within a catchment impact on the hydrology of the catchment as a whole. Palmer et al. (2017) determined, through field measurements and satellite imagery, statistics around two biophysical parameters – leaf area index (LAI), and fraction of photosynthetically active vegetation (fPAR) – which are used in NEE and evapotranspiration (ET) modelling. This knowledge, combined with predictions of how the land cover will change in the future, precipitates the estimation of future carbon storage and water use within the catchment.

Research has shown that land use changes will result in changes to the drivers of earth surface conditions that force General Circulation Models (Cao et al., 2015; Pelletier et al., 2015). Andrews et al. (2017) stated “A radiative forcing arises in response to changes in land cover (e.g. forest to pasture and crops) predominantly because different surface types have different albedos. Forests are generally darker than grasses or croplands and so deforestation tends to increase the Earth's albedo and reflect more solar radiation to space — a negative radiative forcing which causes cooling (e.g. Myhre et al., 2013). However the forcing from changes in land-use is further complicated by its impact on hydrology and non-radiative fluxes (e.g. Brovkin et al., 2006; Betts et al., 2007; Davin et al., 2007; Davin and de Noblet-Ducoudré, 2010; de Noblet-Ducoudré et al., 2012) as well the coincidence of land-cover change and snow cover at higher latitudes (e.g. Betts, 2000; Pitman et al., 2011).” These changes include variations that are linked to surface albedo; that is the earth's ability to absorb or reflect heat energy. For the southern African region, carbon offsets from sequestration may be discounted from the consequences of temperature increases linked to higher albedo. In global change science it is vital to consider surface albedo and surface area of a range of different land cover classes, and to recommend policies that will change albedo to further promote the improvements being offered by carbon off-sets. Thus for each land cover transition the shift in surface albedo should also be considered. Commercial afforestation, IAPs and woody plant encroachment (e.g. *Vachelia karroo*) all result in an increase in the total above-ground woody standing biomass (O'Connor et al., 2014) in this region. In all situations, this is accompanied by an increase in leaf area index (LAI) and possibly a reduction in surface albedo. The higher level of green water in these land cover classes is a good absorber of heat, and this may result in further global heating, possibly discounting the positive consequences of carbon sequestration. In contrast, rural urbanization (which is different from conventional urbanization as dwellings are more widely spaced, and are interspersed with bare soil) may result in higher albedo. Similarly, degraded rangeland, with lower fractional canopy cover, also may have higher albedo (Rotenberg and Yakir, 2010).

This paper builds on previous work (Palmer et al., 2017; Münch et al., 2017; Gwate et al., 2016; Okoye, 2016) by looking at land cover change trends for two grassland-dominated catchments (S50E and T35B) with different land management systems, in the Eastern Cape Province of South Africa. This is a first step in understanding the trend of land cover change on catchment water and carbon fluxes in these catchments. Following an integrated multi-factor analysis of drivers of past land cover change within the catchments, the Land Change Modeller (LCM) in IDRISI was used to simulate future land cover scenarios for the year 2030 and postulate preliminary consequences of this change with respect to carbon storage and water use under each land management system.

## 2 Study area

**Fig. 1:**
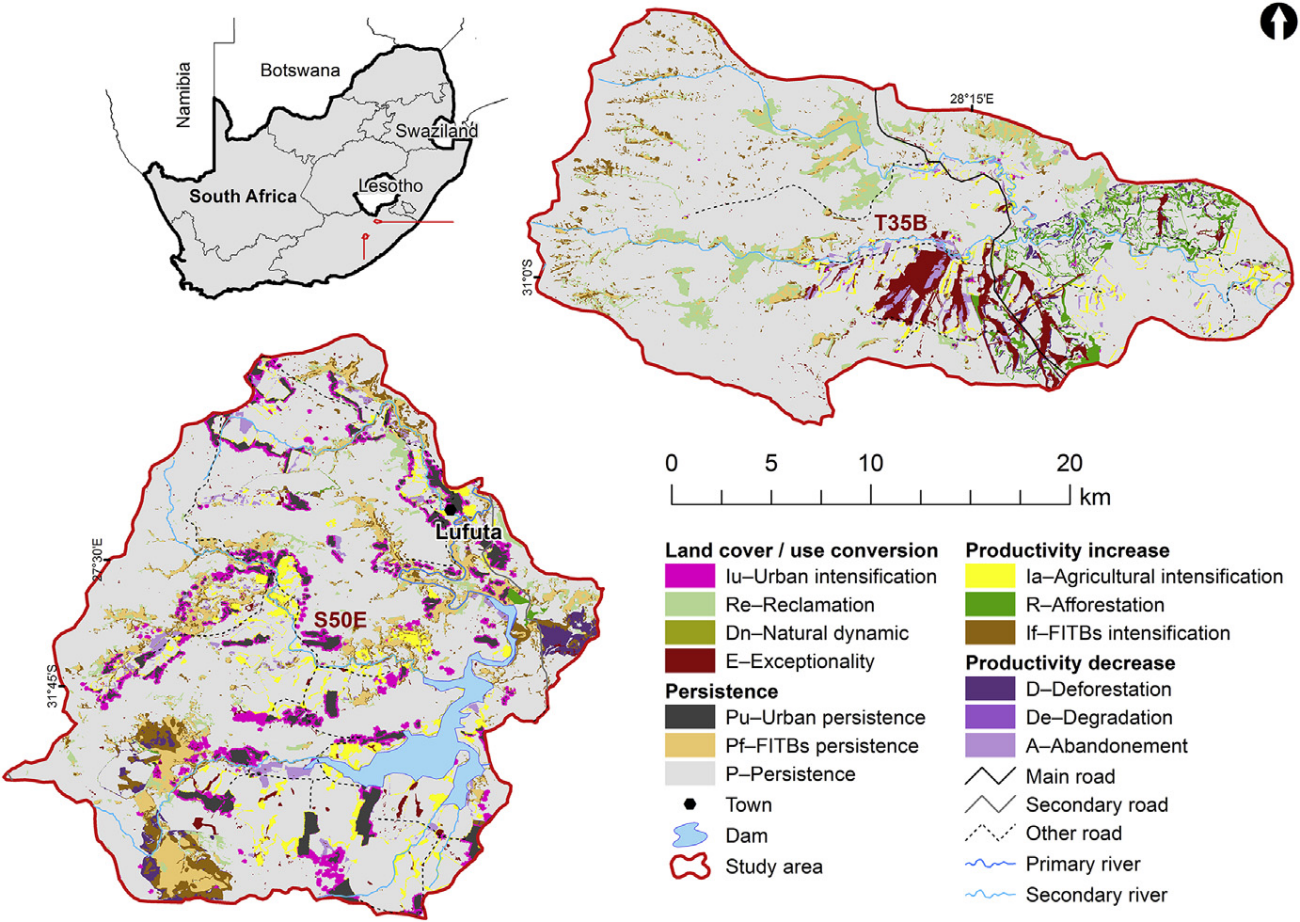
Study area for land cover change analysis.

The S50E and T35B catchments are located in the Eastern Cape Province of South Africa (Fig. 1). In S50E, mixed farming (dualistic farming system) is practiced under communal land tenure arrangements and includes both livestock grazing and crop cultivation (Kakembo, 2001). In T35B, the land tenure is predominantly freehold, and land cover comprises extensive dryland cultivation, commercial afforestation and extensive unimproved grassland for livestock production. However with an average density of 10% (Kotzé et al., 2010), invasion by woody plants, particularly black wattle (*Acacia mearnsii*), silver wattle (*Acacia dealbata*) and poplar (*Populus* spp.), is a major transformer of grasslands and rangeland production. This transformation is aggravated by poor farming practices, including overgrazing, abandonment of cultivation, reduced fire frequency and wood felling that have degraded the vegetation diversity and richness.

The major fluxes of water and carbon in this socio-ecological system occur through livestock and alien trees. Clearing of IAPs in both the catchments is managed by the Department of Environmental Affairs Working for Water (WfW) programme and is premised on increasing water on the landscape in combination with socio-economic development, involving pro-poor interventions (Macdonald, 2004; Oelofse et al., 2016). Clearing IAPs that have higher water use relative to indigenous vegetation (Clulow et al., 2011) is expected to increase the proportion of water to maintain other ecosystem services provided by rangelands (Meijninger and Jarmain, 2014; Van Wilgen et al., 2012). The S50E and T35B catchments are more fully described in Münch et al. (2017).

Land cover maps for change analysis were independently produced from Landsat imagery. An existing national land cover product for 2000 (Van den Berg et al., 2008) was updated through post-classification editing, while a second land cover data set was derived for 2014 using geographic object-based image analysis (GEOBIA) (Okoye, 2016). Theoretical accuracy for land cover change analysis, derived as the product of the accuracies of the independent land cover maps, was lower for T35B (67%) based on lower classification accuracies for 2000 (81%) and 2014 (83%) than for S50E, where accuracies of 83% and 87% respectively for 2000 and 2014 produced a theoretical accuracy of 72% (Münch et al., 2017). Conversion labels were assigned as indicators to describe the transition trajectory identified at each intersection of the two land cover maps (Münch et al., 2017; Okoye, 2016; Benini et al., 2010). Overall observed land cover change in S50E from 2000 to 2014 amounted to 21%, dominated by increased urbanisation and agricultural intensification. However, change could be as high as 42% considering map errors. Persistence and intensification of natural or invaded wooded areas possibly IAPs, were identified as a degradation gradient within the landscape, which amounted to almost 10% of S50E. In some areas, a return to grassland and bare areas signified abandonment and degradation. However, despite a net loss of 5%, grassland still dominates the landscape.

A smaller overall land cover change (18%) was observed in T35B. Plantations increased by 2% (R – Afforestation) through increase in commercial cultivation while grassland was reclaimed (Re) from wooded areas (∼6%), possibly due to eradication of IAPs. Urban areas in T35B remained static as out-migration caused a decline in population (Danuta Hodgson, MSc thesis, unpublished data). Fig. 1 illustrates the two catchments and the land cover change trajectories identified from the land cover change analysis for 2000 to 2014 (Münch et al., 2017; Okoye, 2016).

## 3 Background

**Fig. 2:**
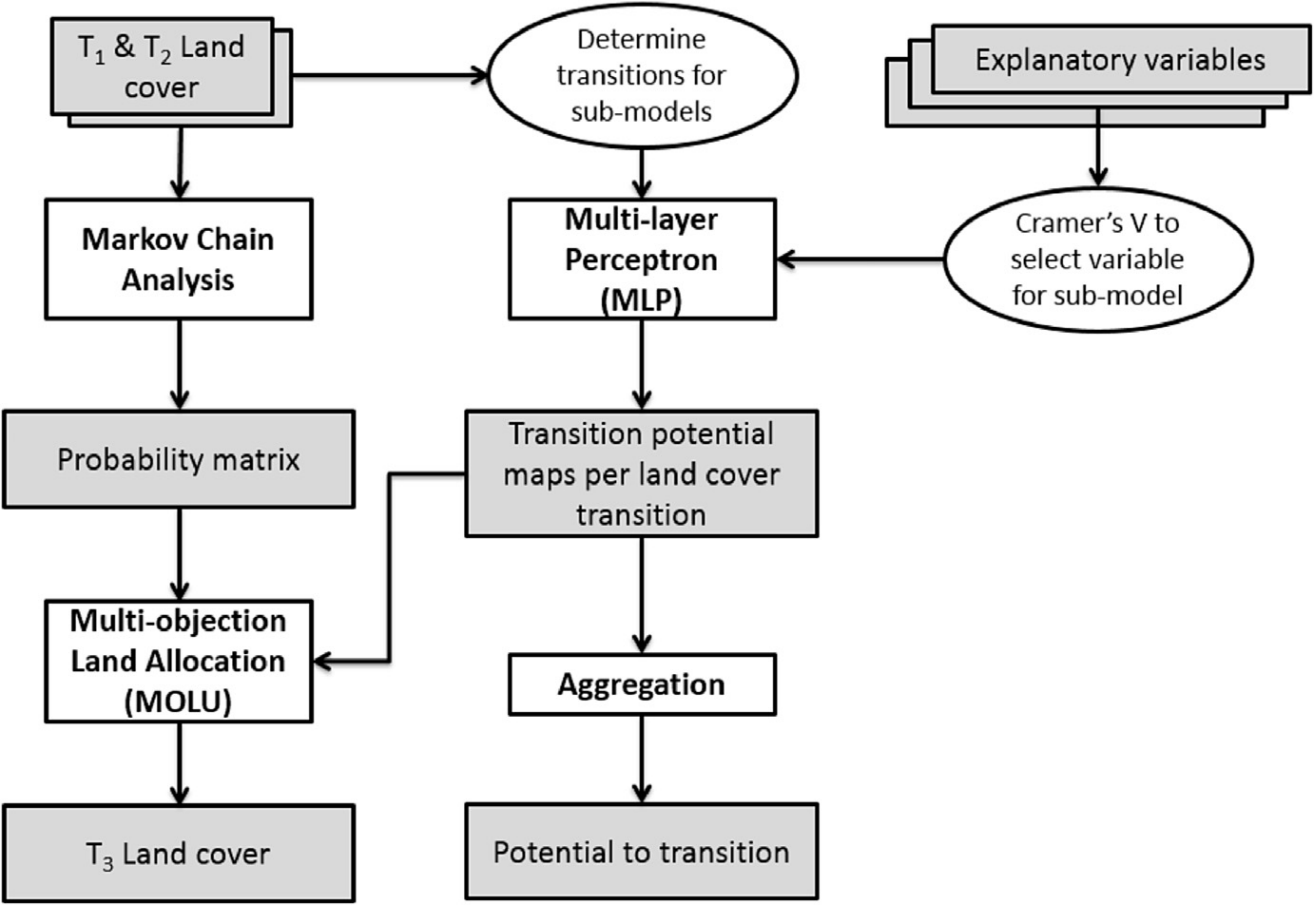
LCM method to predict land cover change.

Most land change models follow a data-driven inductive approach, attempting to draw correlations between a multitude of explanatory factors involved using statistical inferences (Overmars et al., 2007), however a deductive model allows the inclusion of relevant driving factors assumed to have causal influence on LULCC, such as political change or climatic disasters. Land Change Modeller (LCM), an inductive model integrated into IDRISI Terrset 18.08, provides tools for the assessment and projection of land cover change. LCM was developed by Clark Labs in conjunction with Conservation International to provide a suite of tools to address the problems of accelerated land conversion and the analytical needs required in biodiversity conservation (Eastman, 2016). In LCM (Fig. 2), land cover is mapped at two time steps (T_1_ and T_2_) and the patterns and processes of change are estimated and used for model parameterization/calibration (Mas et al., 2014). The approach used in LCM is to analyse changes in land cover between two past time steps (T_1_ and T_2_) and use Multi-layer Perceptron (MLP) with explanatory spatial variables to create transition potential maps. Markov Chain Analysis assigns the probability of change determined by projecting the historic change to the future, which together with transition potential maps, present a land cover scenario for some future data (T_3_). Concurrently, the individual transition potential maps are aggregated to create a map indicating the propensity of the landscape to experience change.

### 3.1 Spatial explanatory variables

Spatial explanatory variables are GIS datasets representing drivers of the observed change (Pérez-Vega et al., 2012) and are typically based on biophysical or socioeconomic criteria. Often used datasets include slope, distance to roads and settlements, land tenure and soil types (Mas et al., 2014) and these driver variables are used to model the historical change process (Eastman, 2016).

The potential explanatory power of a variable can be tested using Cramer's V test where the level of association between GIS datasets representing phenomena thought to be drivers in a particular transition and the land cover in question can be determined. Cramer's V is a quantitative measure of association that ranges from 0.0, indicating no correlation (discarded variable), to 1.0, indicating perfect correlation (excellent potential variable) (Megahed et al., 2015) and although these values are not regarded as definitive, they can help in deciding whether to include an explanatory variable in creating a transition potential map for a transition by examining whether the explanatory variable explains the transition for a particular land cover. According to Eastman (2016), Cramer's V values of 0.15 or higher are ‘useful’ while those with values of 0.4 or higher are ‘good’.

Land cover transitions can be grouped into sub-models if the underlying driver of change is assumed to be the same for each transition (Pérez-Vega et al., 2012). For example, the processes which affect a land cover being changed from forest to urban may be the same as those which affect grassland being converted to urban. This urbanisation transition may be driven by proximity to existing urban areas, proximity to road networks and may have the same topographic driver such as flatter areas are more likely to transition than steep areas. In this example, forest to urban and grassland to urban can be grouped in the same transition or sub-model and the explanatory spatial variables would be the same. Explanatory spatial variables are assigned to each sub-model on the basis of Cramer's V values and the transition potential of each sub-model is determined through a knowledge based approach to machine learning.

### 3.2 Multi-layer perceptron (MLP)

A neural network consists of a number of interconnected nodes which are simple processing elements that respond to the weighted inputs received from other nodes (Atkinson and Tatnall, 1997). The MLP was an advancement in perceptron methods as it is able to separate non-linear data due to it being ‘multi-layer’ and is a popular classification method in remote sensing (Atkinson and Tatnall, 1997). MLP is a feedforward neural network in that data flows in one direction from the input layer through the hidden layers which are sets of computational nodes, to the output layer. The nodes are linked by a web of connections which are applied as a set of weights and a backpropagation algorithm is used to train the network by iteratively spreading the errors from the output layer to the input (Megahed et al., 2015) by adjusting weights so as to minimise the error between the observed and the predicted outcomes (Pérez-Vega et al., 2012). The capability of the model to learn and generalise depends on its architecture (Pérez-Vega et al., 2012) and increasing the number of hidden layers enables the model to learn more complex problems (Atkinson and Tatnall, 1997). The training performance is assessed by a precision value expressed in percent and networks that are too small cannot identify the internal structure of the data and produce lower performance accuracies whereas too large networks overfit the training data (Pérez-Vega et al., 2012).

The aim of the training is to build a model of the data generating process so that network outputs can be predicted from unseen inputs. The network output is then compared with the desired output, the error is computed and then back-propagated through the network to adjust weights (Atkinson and Tatnall, 1997). Large quantities of data are often required for training (Atkinson and Tatnall, 1997) and thus a small sample of training sites is unlikely to result in an accurate model.

In the IDRISI MLP, half of the training data are randomly selected for learning and half for validation. After the MLP has been trained, validation data are used to calculate a "skill measure" (computed as the accuracy of transition prediction minus the accuracy expected by chance) (Mas et al., 2014). The MLP thus creates time-specific transition potential maps for each of the sub-models which are expressions of time-specific potential for change (Eastman, 2016). However, further steps are required to use this information to predict future land cover classes and also the potential for each cell to either persist or transition between land covers.

### 3.3 Markov chain

Markov chain analysis is a stochastic modelling approach which has been used extensively for land cover change modelling (Fathizad et al., 2015). It assumes that the probability of a system being in a certain state at a certain time can be determined if its state at a prior time is known with the assumption that rates of change observed during the calibration period (T_1_ to T_2_), will remain the same during the simulation period (T_2_ to T_3_). Through cross-tabulation of land cover (Kamusoko et al., 2009) Markov chain analysis determines the amount of land cover change that will occur to the future date (Eastman, 2016). In LCM transition probability maps are produced using either logistic regression, MLP trained by backpropagation or a machine learning approach (Mas et al., 2014). This provides a probability estimate for each pixel to either be transformed to another land cover or to persist and be calibrated to an annual time step (Kamusoko et al., 2009).

### 3.4 Future scenarios

LCM produces two predictors of future land cover: soft prediction and hard prediction. Soft prediction, or potential to transition, is a continuous mapping of vulnerability to change (Eastman, 2016). It is calculated by aggregating all the transition potentials and provides an indication of the degree to which the areas have the right conditions to precipitate change. The soft predictor thus provides a likelihood of a cell to experience land cover change without providing an indication as to what the new land cover will be.

The hard prediction procedure used by LCM is based on TerrSet's multi-objective land allocation (MOLA) module. MOLA determines a compromise solution by maximizing the suitability of lands for each objective given the assigned weights (Eastman, 2016). Land allocation conflicts are resolved by allocating the cell to the objective (land cover class) for which its weighted transition potential is highest based on a minimum distance to ideal point rule using the weighted ranks (Houet and Hubert-Moy, 2006). Finally, the transition probability matrix derived from the Markov chain analysis determines how much land is allocated to a class over, T_3_ – T_2_, an n-year period.

In LCM, change is thus modelled through MLP using mathematics and explanatory spatial variables in a trends driven approach (Pérez-Vega et al., 2012). Spatial analysis of land cover change using the explanatory variables identifies: 1) maps of the transition potential for each identified land cover transition, 2) a transition potential map indicating the likelihood of each location in the study area to experience change and 3) a scenario land cover map for a selected future date (T_3_).

## 4 Methods

Land cover maps at 30 m pixel resolution (Okoye, 2016), for T_1_ (2000) and T_2_ (2014) were used to create 1) transition potential maps for each transition, 2) a projected potential for transition map and 3) a predicted land cover map for 2030 (T_3_) for S50E and T35B. An identical land cover legend consisting of eight land cover classes was used for each time step (Table 1).

**Table 1:** Land cover legend developed by Münch et al. (2017).

Abbreviation	Description
UG	Unimproved (degraded/natural) grassland
FITBs	Forest indigenous, thicket bushlands, bush clumps, high fynbos
BRS	Bare rock and soil (natural)
Wb	Water bodies
Wl	Wetlands
CLS	Cultivated land
FP	Forest plantations (clear-felled, pine spp., other/mixed spp.)
UrBu	Urban/built-up (residential, formal township)

Following Münch et al. (2017), trajectories of land cover change describing both change and persistence were identified and each possible transition of land cover between T_1_ and T_2_ was labelled (Table 2). Following Pérez-Vega et al. (2012) land cover transitions with common underlying drivers of change were grouped into sub-models. Trajectories of land cover change, identifiable at data resolution (30 m) were also labelled (Table 2) as representing (1) intensification - the transition of a lower intensity to a higher intensity usage; (2) afforestation - the planting of commercial trees; (3) deforestation - the clearance of trees; (4) reclamation, degradation and abandonment related to conversion to grassland and bare areas; (5) natural dynamics - seasonal conversions not explained through anthropogenic change; and (6) exceptionality - associated with potential map errors (Münch et al., 2017).

**Table 2:** Land cover conversion labels related to land cover change and drivers.

Class Label		2014							
		UG	FITBs	BRS	Wb	Wl	CLs	FPs	UrBu
2000	UG	P	IF (1)	De (4)	Dn (5)	Dn (5)	Ia (1)	R (2)	Iu (1)
	FITBs	Re (4)	P	Re (4)	E (6)				
	BRS	Dn (5)	IF (1)	P					
	Wb			Dn (5)	P				E (6)
	Wl				Dn (5)	P			Iu (1)
	CLs	A (4)		A (4)	E (6)	E (6)	P		
	FPs	D (3)		D (3)			Ia (1)	P	
	UrBu	A (4)		A (4)				R (2)	P

Of particular importance are areas where another land cover class has potentially been replaced by IAPs (FITB intensification) and where forests (indigenous or alien) and other woody areas have disappeared or been removed (reclamation, deforestation). A change from any other land cover class (with the exception of waterbodies and wetlands) were labelled Iu: Urban intensification. It was not possible to determine change in the intensity of agricultural activities due to image resolution, but conversion to agricultural practices was identified (agricultural intensification). Although persistence (P) — where no land cover change has occurred — can be considered a trajectory, it cannot be considered a transition and thus trajectories representing persistence are ignored by LCM. In reality, not all possible transitions occurred between 2000 and 2014 in S50E and T35B. Due to the low user's and producer's accuracy for LC classes bare soil (BRS) and wetlands (Wl), Münch et al. (2017) labelled all transitions involving these classes as potential classification error. Table 3 displays the labels, transitions and description for each sub-model. Small transitions (less than 10 ha) have been removed from the analysis to exclude exceptionalities.

**Table 3:** Transition sub-models and descriptors for catchment S50E and T35B.

Transition sub-model	Description	Land cover transitions*^+^
**If: FITBs intensification (↑FITBs)**	Woody natural and artificial vegetation substitutes previous land cover	UG to FITBs; **FP to FITBs**; CLS to FITBs
**Ia: Agricultural intensification (↑Agric)**	Agricultural activities substitute previous land cover	UG to CLS; FITBs to CLS; Wb to CLS*; Wl to CLS; UrBu to CLS; *FP to CLS*^*+*^
**Iu: Urban intensification (↑Urban)**	Urban activities substitute previous land cover	UG to UrBu; CLS to UrBu*; FITBs to UrBu
**R: Afforestation (↑Forest)**	Other land covers are converted to plantations	UG to FP; FITBs to FP; *WL to FP*^*+*^*; CLS to FP*^*+*^
**D: Deforestation (↓Forest)**	Plantations converted to other land covers	FP to UG; FP to BRS*; *FP to Wl*^*+*^
**A: Abandonment (Abandon)**	Urban and agricultural areas converted to grassland and bare areas	CLS to UG; UrBu to UG; *CLS to Wl*^*+*^
**Dn: Natural dynamic (Natural)**	Areas where natural changes occurred	UG to Wb; UG to Wl; Wb to UG; Wl to UG; *FITBs to Wl*^*+*^
**De: Degradation (Degrade)**	Shrub area converted to grassland and bare areas	UG to BRS
**Re: Reclamation (Reclaim)**	Woody natural and artificial vegetation areas converted to grassland and bare area	FITBs to UG

*Bold text shows transitions which occurred in S50E only.

^+^*italics* show transitions that occurred only in T35B.

In choosing explanatory variables, the processes producing land cover change need to be visualised after which a spatial dataset of the particular process must be sourced at appropriate spatial resolution. GIS data sets were identified to describe the potential transitions, geo-processing was performed to represent the particular process and abbreviations were assigned to each processed spatial dataset. In addition to geographical parameters, Evidence likelihood (EV) which calculates the relative frequency of pixels which belong to the different classes within the areas of change, is recommended where there are low Cramer V values (Eastman, 2016).

Derived biophysical and anthropogenic datasets were tested for their suitability using Cramer's V where higher values represent stronger relationships between the variable and a particular transition with values higher than 0.4 regarded as good (Megahed et al., 2015).

Within the context of the communal/traditional farming methods practiced, proximity to parts of the landscape already impacted by people may be considered as potential drivers for degradation. For IAP intensification, infestation is more likely to occur in those areas close to existing infestation through the process of seed dispersal. Similarly afforestation is more likely to occur in those areas close to existing plantations since infrastructure is already in place to support this. Topographic variables can be considered as having a potential constraining or flourishing effect of certain transitions. For example, water bodies will not expand into areas with a slope and certain vegetation may not grow higher than a specified altitude. Finally, vegetation distribution is influenced by access to water and thus the Euclidean distance from rivers may be used as a proxy for water availability.

## 5 Results

### 5.1 Explanatory spatial variables

The variables selected for each transition sub-model are shown in Tables 4 and 5 for S50E and T35B respectively. Overall Cramer's V values are shown in Table 6 with Cramer's V values for individual land cover classes given in Table 7.

**Table 4:** Sub-models included in MLP for S50E with associated explanatory variables and performance indicators.

Sub-model	Explanatory variables	Transition/Persistence Class	Minimum cells transitioned/persisted	Class skill measure (ratio)	Sub-model Accuracy (%)	Sub-model skill	RMS	
							Training	Testing
If: FITBs intensification	*Elev* *Slope* *D_FP* *D_FITBs* *D_rd* *D_res*	UG to FITBs	1846	0.4416	69.36	0.6324	0.2692	0.2733
		CLS to FITBs		0.7959				
		FP to FITBs		0.7587				
		Persistence: UG	7918	0.4644				
		Persistence: CLS		0.6789				
		Persistence: FP		0.6601				
Ia: Agricultural intensification	*Elev* *Slope* *Asp* *D_res* *EV*	UG to CLS	32	0.8693	50.34	0.4482	0.2452	0.2530
		FITBs to CLS		−0.1111				
		Wb to CLS		−0.1111				
		Wl to CLS		0.3519				
		UrBu to CLS		−0.0317				
		Persistence: UG	508	0.6732				
		Persistence: FITBs		0.7222				
		Persistence: Wb		1.0000				
		Persistence: Wl		0.2361				
		Persistence: UrBu		0.5238				
Iu: Urban intensification	*Elev* *D_FITBs* *D_rd* *D_res*	UG to UrBu	1875	−0.1048	54.34	0.4521	0.3196	0.3197
		FITBs to UrBu		0.8399				
		CLS to UrBu		0.4775				
		Persistence: UG	30778	0.4189				
		Persistence: FITBs		0.6048				
		Persistence: CLS		0.4617				
R: Afforestation	*Elev* *Asp* *D_FP* *D_FITBs*	UG to FP	342	0.5400	49.39	0.3252	0.3786	0.3856
		FITBs to FP		0.4865				
		Persistence: UG	30778	0.4615				
		Persistence: FITBs		−0.1686				
D: Deforestation	*Elev* *Asp* *D_riv* *D_rd*	FP to UG	137	0.1269	66.51	0.4976	0.3804	0.3972
		FP to BRS		0.8433				
		Persistence: FP	7918	0.5192				
A: Abandonment	*Elev* *Slope* *Asp*	CLS to UG	503	0.1926	37.45	0.1660	0.4150	0.4205
		UrBu to UG		0.5060				
		Persistence: CLS	20948	0.0985				
		Persistence: UrBu		−0.1390				
Dn: Natural dynamic	*Elev* *Slope* *Asp* *D_riv*	UG to Wb	32	0.3246	39.13	0.2899	0.3131	0.3196
		UG to Wl		0.1979				
		Wb to UG		−0.1667				
		Wl to UG		−0.1667				
		Persistence: UG	162	0.5702				
		Persistence: Wb		0.9222				
		Persistence: Wl		0.2708				
De: Degradation	*Elev* *Slope* *Asp* *D_riv* *D_res* *EV*	UG to BRS	409	0.4314	69.76	0.3951	0.3995	0.4457
		Persistence: UG	252574	0.3592				
Re: Reclamation	*Elev* *Slope* *D_riv* *D_res* *EV* *D_FITBs*	FITBs to UG	13843	0.0866	62.47	0.2494	0.4723	0.4750
		Persistence: FITBs	30778	0.4137

**Table 5:** Sub-models included in MLP for T35B with associated explanatory variables and performance indicators.

Sub-model	Explanatory variables	Transition/Persistence Class	Minimum cells that transitioned/persisted	Class skill measure (ratio)	Sub-model Accuracy (%)	Sub-model skill	RMS	
							Training	Testing
If: FITBs intensification	*Elev*	UG to FITBs	222	0.6751	67.89	0.5719	0.3195	0.3274
	*Slope*	CLS to FITBs		0.4234				
	*D_FP*							
	*D_rd*	Persistence: UG	19736	0.2072				
	*D_riv*	Persistence: CLS		0.5385				
	*EV*							
Ia: Agricultural intensification	*Elev* *Slope* *D_rd* *D_riv* *EV*	UG to CLS	122	0.3464	64.98	0.6108	0.2144	0.2197
		FITBs to CLS		0.9668				
		FP to CLS		0.9454				
		Wl to CLS		0.6633				
		UrBu to CLS		0.4359				
		Persistence: UG	309	0.4362				
		Persistence: FITBs		0.6649				
		Persistence: FP		0.4444				
		Persistence: Wl		0.6481				
		Persistence: UrBu		0.5214				
Iu: Urban intensification	*Elev* *Slope* *D_FP* *D_rd* *D_riv*	UG to UrBu	187	0.9028	82.84	0.7712	0.3305	0.3274
		FITBs to UrBu		0.8996				
		Persistence: UG	7586	0.5362				
		Persistence: FITBs		0.7391				

R: Afforestation	*Elev* *D_FP* *D_rd* *EV*	UG to FP	569	0.9257	89.28	0.8775	0.1657	0.1734
		FITBs to FP		0.9102				
		Wl to FP		0.9678				
		CLS to FP		1.0000				
		Persistence: UG	1996	0.7104				
		Persistence: FITBs		0.9246				
		Persistence: Wl		0.7771				
		Persistence: CLS		0.8115				
D: Deforestation	*Elev* *Asp* *EV*	FP to UG	437	0.3411	53.02	0.2953	0.4302	0.4342
		FP to Wl		0.7867				
		Persistence: FP	23904	−0.2535				
A: Abandonment	*Elev* *D_FP* *EV*	CLS to UG	387	0.2734	42.69	0.2837	0.3454	0.3453
		UrBu to UG		0.5833				
		CLS to Wl		0.5283				
		Persistence: CLS	309	−0.2500				
		Persistence: UrBu		0.2695				
Dn: Natural dynamic	*Elev* *Slope* *Asp*	FITBs to Wl	155	0.5143	36.47	0.2739	0.3045	0.3108
		UG to Wl		−0.1429				
		Wb to UG		0.4603				
		Wl to UG		−0.1429				
		Persistence: UG	65	0.3364				
		Persistence: Wb		0.5357				
		Persistence: FITBs		0.6327				
		Persistence: Wl		−0.1429				
De: Degradation	*Asp* *EV* *D_FP* *D_FITBs*	UG to BRS	605	0.3510	71.16	0.4233	0.4474	0.4472
		Persistence: UG	306061	0.4983				
Re: Reclamation	*Elev* *Slope* *D_rd* *D_FP* *EV*	FITBs to UG	26674	0.0168	54.67	0.0935	0.4929	0.4956
		Persistence: FITBs	7586	0.1695

**Table 6:** Description of potential explanatory variables and their overall Cramer's V value. Cramer's V values for S50E and T35B are shown in bold and italics respectively.

Variable	Elevation	Aspect	Slope	Distance from FP (2000)	Distance from FITBs (2000)	Distance from rivers	Distance from roads	Distance from residential areas	Evidence likelihood
**Abbreviated**	*Elev*	*Asp*	*Slope*	*D_FP*	*D_FITBs*	*D_riv*	*D_rd*	*D_res*	*EV*
**Data source**	USGS SRTM 1 Arc-Second (USGS, 2004)			Land cover 2000 (Münch et al., 2017)		NGI vector data (National Geo-Spatial Information).			
**Geo processing**		Aspect computed for Elevation dataset	Slope computed from Elevation dataset	Extracted LC Class FP; Euclidian distance from FP	Extracted FITBs; Euclidian distance from FITBs	Rasterize; Euclidian distance from all rivers	Rasterize; Euclidian distance from all roads	Rasterize; Euclidian distance from residential areas	
**Scale**	∼30 m cell resolution			Cell resolution 30 m		1: 50 000 vector scale converted to 30m cell resolution			
**CRAMER V**									
**Overall**	**0.2675**	**0.2134**	**0.259**	**0.1997**	**0.1812**	**0.0978**	**0.1719**	**0.2298**	
	*0.2065*	*0.0887*	*0.1666*	*0.2747*	*0.087*	*0.1047*	*0.128*		*0.4083*

**Table 7:** Potential explanatory variables based on Cramer's V values. ‘Good’ values are considered to be higher than 0.4 whilst ‘useful’ values are higher than 0.15. Values for S50E are bold and T35B is shown in italics.

Variable	Elevation	Aspect	Slope	Distance from FP (2000)	Distance from FITBs (2000)	Distance from rivers	Distance from roads	Distance from residential areas	Evidence likelihood
CRAMER'S V									
**Overall**	**0.2675**	**0.2134**	**0.259**	**0.1997**	**0.1812**	**0.0978**	**0.1719**	**0.2298**	
	*0.2065*	*0.0887*	*0.1666*	*0.2747*	*0.087*	*0.1047*	*0.128*		*0.4083*
**UG**	**0.4366**	**0**	**0**	**0**	**0**	**0**	**0**	**0**	
	*0*	*0*	*0*	*0*	*0*	*0*	*0*		*0*
**FITBs**	**0.199**	**0.2026**	**0.3798**	**0.2029**	**0.2448**	**0.1304**	**0.2404**	**0.3121**	
	*0.4444*	*0.1074*	*0.3037*	*0.4971*	*0.1128*	*0.194*	*0.2657*		*0.6264*
**BRS**	**0.0288**	**0.1194**	**0.098**	**0.2671**	**0.3475**	**0.1615**	**0.0923**	**0.1725**	
	*0.1728*	*0.1861*	*0.1401*	*0.1848*	*0.2032*	*0.1216*	*0.0687*		*0.2583*
**Wb**	**0.4261**	**0.0334**	**0.0134**	**0.0228**	**0.0181**	**0.0108**	**0.0243**	**0.0234**	
	*0.0202*	*0.0383*	*0.0084*	*0.0317*	*0.0114*	*0.0069*	*0.0464*		*0.0111*
**Wl**	**0.0199**	**0.5539**	**0.5593**	**0.124**	**0.1589**	**0.0759**	**0.2038**	**0.1154**	
	*0.0474*	*0.0104*	*0.0206*	*0.0318*	*0.0127*	*0.0163*	*0.0203*		*0.2431*
**CLS**	**0.4139**	**0.0169**	**0.0236**	**0.0203**	**0.0183**	**0.047**	**0.0106**	**0.0147**	
	*0.1693*	*0.072*	*0.1928*	*0.1217*	*0.0311*	*0.0492*	*0.0359*		*0.2369*
**FP**	**0.163**	**0.1952**	**0.4151**	**0.2239**	**0.3385**	**0.2047**	**0.1291**	**0.2892**	
	*0.3678*	*0.1322*	*0.3553*	*0.3004*	*0.1014*	*0.195*	*0.1294*		*0.7176*
**UrBu**	**0.2087**	**0.0321**	**0.0306**	**0.4122**	**0.061**	**0.0211**	**0.0779**	**0.0734**	
	*0.3472*	*0.0654*	*0.1715*	*0.6749*	*0.0777*	*0.1677*	*0.3104*		*0.7248*

### 5.2 Transition potential and prediction

**Fig. 3:**
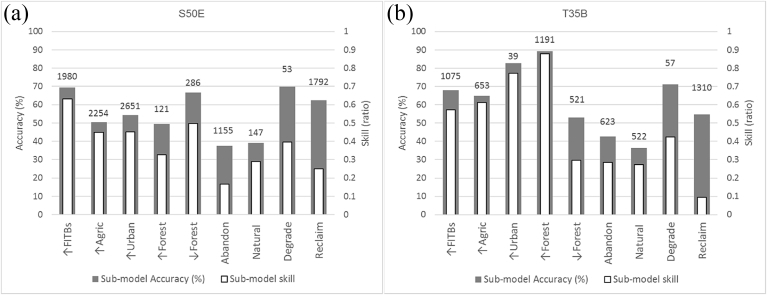
Sub-model accuracy and skill measure from MLP for (a) S50E and (b) T35B. Figures above bars depict the number of pixels in each submodel.

**Fig. 4:**
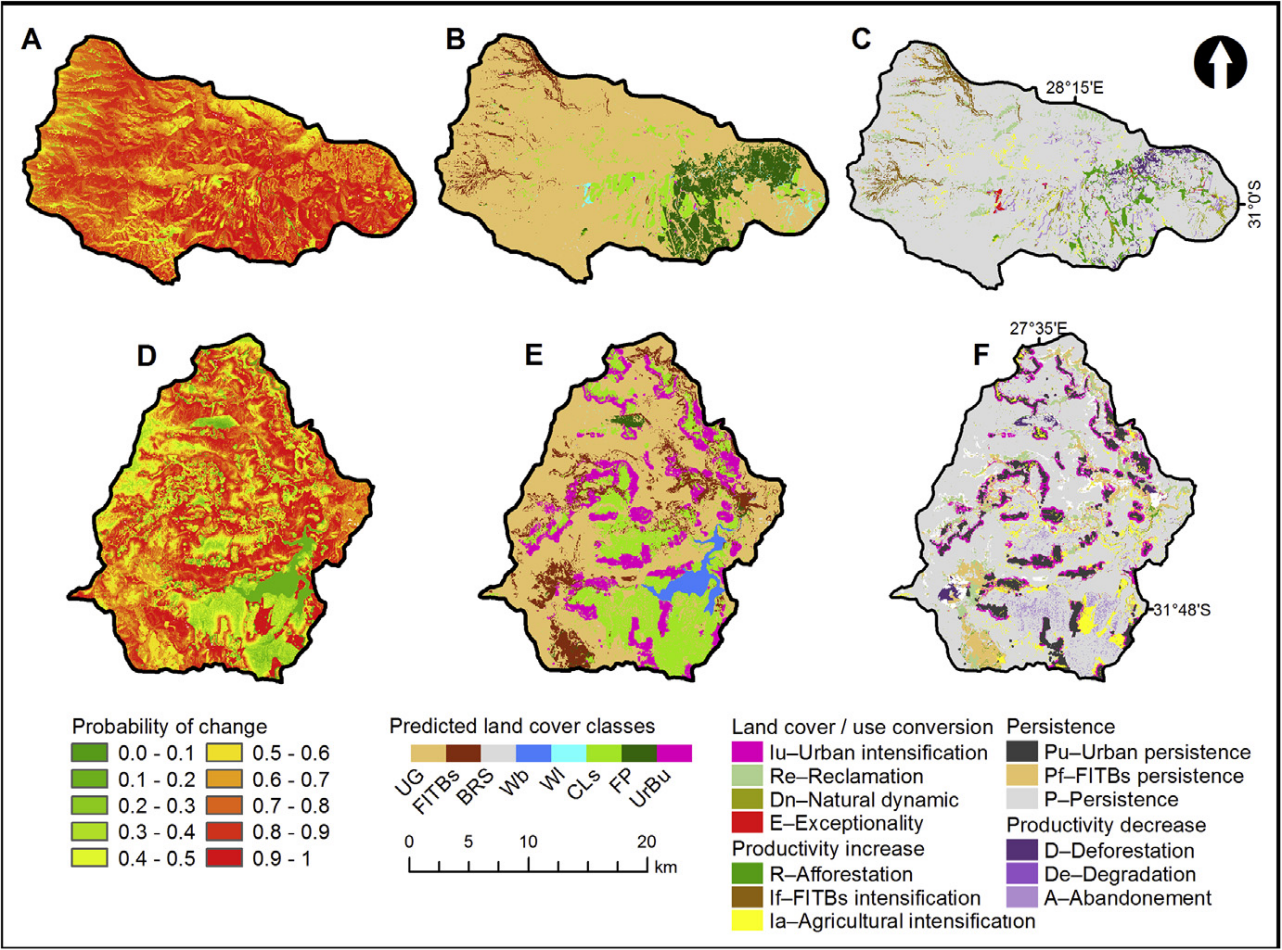
Maps of results showing: propensity to change in T35B (A) and S50E (D), the land cover classes predicted for 2030 in T35B (B) and S50E (E), and land cover conversion/persistence for T35B (C) and S50E (F).

The skill measure and accuracy rate of each sub-model calculated through MLP, summarized in Fig. 3, are recorded in Table 4 (S50E) and Table 5 (T35B) and shown as maps in Fig. 4. The skill measure is based on the 2000 and 2014 land cover maps and compares the number of correct predictions, minus those attributable to random guessing, to that of a hypothetical set of perfect predictions. Thus the skill measure is not an evaluation of future performance of the model but rather a gauge of how well the explanatory variables explained change in the past.

The accuracy and skill measure reveal a wide disparity between the levels of confidence in model predictions for different transitions. In S50E, the accuracy varies between 37 and 70 percent, with a correlation of 0.5 between accuracy and number of pixels involved in transition (Table 4). Lowest accuracy is associated with Abandonment (A) which also has the lowest skill measure. This is followed by Natural dynamic (Dn) with less than 40% accuracy and skill measure of less than 0.3. Degradation (De) has a high accuracy due to the large number of pixels involved in this sub-model (persistence of UG), but a low skill measure. These anomalies may be explained by the low user's and producer's accuracy for LC classes Wetlands (Wl) (in sub-model Dn) and Bare rock and soil (BRS) (in sub-model De) affecting the MLP (Münch et al., 2017) but may also be an indication that change is not totally controlled by the drivers used in the model. Low accuracies amongst those transitions which involve a small number of pixels should be regarded as being of low importance.

Afforestation (R) and Urban intensification (Iu) in T35B have accuracies higher than 80% and matching high skill measures. Natural dynamics (Dn) has the lowest accuracy in T35B similar to S50E. Re (reclamation from FITBs) has the lowest skill score of less than 0.1 based on the low class skill ratio for the FITBs to UG transition.

The probability of a land cover persisting (the diagonal highlighted by *) and of each class transitioning to every other class from the Markov matrix are presented in Table 8.

**Table 8:** Markov matrix probability of land covers in S50E (bold) and T35B (italics) transitioning or persisting (*) from 2014 to 2030. Note land cover abbreviations are given in Table 1.

	UG	FITBs	BRS	Wb	Wl	CLS	FP	UrBu
UG	0.80*	**0.05**	**0.00**	**0.00**	**0.00**	**0.07**	**0.00**	**0.08**
	0.91*	*0.03*	*0.00*	*0.00*	*0.01*	*0.02*	*0.03*	*0.00*
FITBs	**0.34**	0.58*	**0.00**	**0.00**	**0.00**	**0.02**	**0.02**	**0.04**
	*0.82*	0.10*	*0.00*	*0.00*	*0.01*	*0.03*	*0.03*	*0.00*
BRS	**0.43**	**0.05**	0.00*	**0**	**0.00**	**0.02**	**0.01**	**0.49**
	*0.25*	*0.00*	0.00*	*0*	*0.01*	*0.11*	*0.62*	*0.00*
Wb	**0.03**	**0.00**	**0**	0.93*	**0**	**0.04**	**0**	**0.00**
	*0.56*	*0.01*	*0.00*	0.07*	*0.16*	*0.13*	*0.06*	*0.00*
Wl	**0.52**	**0.01**	**0.00**	**0.00**	0.00*	**0.43**	**0**	**0.03**
	*0.68*	*0.01*	*0.00*	*0.00*	0.06*	*0.12*	*0.13*	*0.00*
CLS	**0.11**	**0.03**	**0.00**	**0.00**	**0.00**	0.84*	**0.00**	**0.03**
	*0.24*	*0.01*	*0.00*	*0.00*	*0.03*	0.69*	*0.02*	*0.00*
FP	**0.34**	**0.42**	**0.00**	**0**	**0.00**	**0**	0.24*	**0.00**
	*0.16*	*0.00*	*0.00*	*0*	*0.02*	*0.01*	0.82*	*0.00*
UrBu	**0.03**	**0.00**	**0.00**	**0**	**0**	**0.05**	**0.02**	0.92*
	*0.46*	*0.04*	*0.00*	*0.00*	*0.02*	*0.27*	*0.02*	0.19*

Table 9 shows the modelled land cover change based on the proportion of the study area. The loss and gain per class is also recorded with the net loss per land cover class indicated. In S50E, the probability of UG persisting is approximately 80% with the highest probability of UG being lost are to FITBs (∼4.5%), CLS (∼6.6%) and UrBu (∼8.3%), thus FITBs intensification (If), agricultural intensification (Ia) and urban intensification (Iu) are at the expense of grasslands, constituting ∼12% of the catchment (5,283 ha) as shown in Table 9. The probability of 34% FITBs loss to UG (Table 8), possibly due to alien invasive clearing programs, may seem high, but in reality the number of pixels that can in fact transition are limited and the change represents only 4% (1800 ha) of the total area (44,640 ha) in 2030 (Table 9). The probability of persistence of FP is low (24%) with a likelihood of transition to FITBs (42%) and UG (34%), which clearly reflects the changes from 2000–2014. Classes Wl and BRS also show a very low probability of persisting.

**Table 9:** Modelled land cover change as a percentage of the study area for S50E (bold) and T35B (italics), * denotes persistence.

Change	UG	FITBs	BRS	Wb	Wl	CLs	FP	UrBu	Total 2014	Loss	Net
UG	44.7*	**3.2**	**0.1**	**0.1**	**0.1**	**4**	**0.1**	**4.7**	**56.9**	**12**	***−4.8***
	72.7*	*2.7*	*0.1*	*0*	*0.5*	*1.3*	*2.6*	*0.1*	*79.9*	*7.2*	*−0.2*
FITBs	**4**	5.5*	**0**	**0**	**0**	**0.3**	**0.2**	**0.5**	**10.5**	**5.1**	***−0.6***
	*3.3*	0.4*	*0*	*0*	*0*	*0.1*	*0.1*	*0*	*4*	*3.6*	*−0.9*
BRS	**0**	**0**	0.1*	**0**	**0**	**0**	**0**	**0**	**0.1**	**0.1**	**0.1**
	*0*	*0*	0.2*	*0*	*0*	*0*	*0*	*0*	*0.2*	*0*	*0.1*
Wb	**0.1**	**0**	**0**	2.6*	**0**	**0.1**	**0**	**0**	**2.9**	**0.3**	***−0.2***
	*0*	*0*	*0*	0*	*0*	*0*	*0*	*0*	*0*	*0*	*0*
Wl	**0**	**0**	**0**	**0**	0*	**0**	**0**	**0**	**0.1**	**0.1**	***−0.01***
	*0.8*	*0*	*0*	*0*	0.1*	*0.1*	*0.2*	*0*	*1.2*	*1.1*	*−0.3*
CLs	**2.2**	**0.5**	**0**	**0**	**0**	15*	**0**	**0.7**	**18.2**	**3.4**	**1.7**
	*1.5*	*0.1*	*0*	*0*	*0.2*	4.3*	*0.2*	*0*	*6.2*	*1.9*	*−0.2*
FP	**0.6**	**0.7**	**0**	**0**	**0**	**0**	0.4*	**0**	**1.8**	**1.4**	***−1.1***
	*1.3*	*0*	*0*	*0*	*0.1*	*0*	6.8*	*0*	*8.3*	*1.5*	*1.5*
UrBu	**0.3**	**0.1**	**0**	**0**	**0**	**0.6**	**0**	8.5*	**9.5**	**1**	**4.9**
	*0.1*	*0*	*0*	*0*	*0*	*0.1*	*0*	0.1*	*0.2*	*0.1*	*0*
Total 2030	**52.1**	**9.9**	**0.2**	**2.7**	**0.1**	**20**	**0.7**	**14.4**			
	*79.7*	*3.1*	*0.3*	*0*	*0.9*	*6*	*9.8*	*0.2*			
Gain	**7.4**	**4.4**	**0.1**	**0.1**	**0.1**	**5.1**	**0.3**	**5.9**		**23**	
	*7*	*2.7*	*0.1*	*0*	*0.8*	*1.7*	*3*	*0.1*		*16*	
Change per year										**1.5**	
										*1*	

In T35B, the probability of UG persisting is over 90% with the highest probability of UG being lost are to FITBs (∼3.3%) and FP (3.2%) (Table 8).

Based on the cross tabulation of land cover classes (Table 9), it was determined that the total change (gain and loss) in the landscape for catchment S50E over all land cover classes was 23% for predicted period 2014 to 2030, compared with 21% for the period between 2000 and 2014 (Münch et al., 2017), assuming a similar map accuracy for the modelled map. Since the future scenario model mimics patterns of past measured change, the change intensity, defined as the change per year, remained constant at 1.5% per year for S50E. UG, the largest class, also has the largest loss, though this relatively large dormant class, shows a higher change intensity during the modelled period with the loss intensity increasing from 1.27% to 1.34%. In contrast to the measured change, a net loss was modelled for FITBs. However, the predicted loss falls within the 30% hypothetical error in landscape transition ascribed to error propagation from contributing land cover maps calculated by Münch et al. (2017). Net change in FITBs for 2000 to 2014 varied between −0.5% to +1% of total catchment area. In T35B, the total change (gain and loss) in the landscape over all land cover classes was only 15.5% for prediction period 2014 to 2030, compared with 18.2% for the period between 2000 and 2014 (Münch et al., 2017). The change intensity decreased from 1.3% to less than 1% for T35B. FP showed a small net gain. Intensification of FITBs were modelled in the upper reaches of the Pot River and Little Pot. While FITBs systematically targets UG in transition (If), clearing of FITBs also systematically results in UG (Re), though possibly degraded, with a net loss of FITBs over the period. Afforestation (increased FP) is the strongest trajectory in T35B showing a net gain of 1.5% with FP targeting Wl. This transition may be the result of the low accuracy of the Wl class in the 2014 input land cover dataset.

### 5.3 Evaluating land cover future scenario

Since the result of this model is a future scenario, typical land cover validation methods cannot be employed since T_3_ is a future time step. Other indicators are thus required to assess the prediction. While visual examination reveals spatial patterns, it is subjective and can be misleading. Pontius and Millones (2011) suggest that disagreement in land cover maps can be attributed to randomness based on: 1) random distribution of the quantity of each land cover class (quantity disagreement), and 2) random spatial allocation of the land cover classes (allocation disagreement). In addition, in this study, the disagreement could also be attributed to errors in the land cover prediction model. However, the disagreement statistics can provide an indication of the quality of the future scenario map. The disagreement budget between the actual land cover maps 2014 (T_2_) and 2000 (T_1_), as well as between modelled land cover classes (T_3_) and 2014 land cover classes (T_2_), is provided in Table 10. Quantity difference is defined as the amount of difference between the T_2_ map and a comparison map where the proportions of the classes do not match. Allocation disagreement occurs where the quantity per class remains the same but the spatial distribution of the class changes and can be separated into exchange and shift. Exchange describes the transition between the misallocated classes. Shift refers to the difference remaining after subtracting quantity difference and exchange from the overall difference (Pontius and Santacruz, 2014).

**Table 10:** Comparison between transitions for 2000 to 2014 (T1–T2) and 2014 to 2030 (T2–T3) for S50E and T35B.

Class	S50E						T35B					
	2000–2014			2014–2030			2000–2014			2014–2030		
	Quantity	Exchange	Shift	Quantity	Exchange	Shift	Quantity	Exchange	Shift	Quantity	Exchange	Shift
UG	4.5	***8.9***	4.0	4.8	***12.0***	2.8	4.3	***9.3***	2.7	0.2	***11.6***	2.5
FITBs	0.8	5.9	1.8	0.6	7.5	1.4	4.3	4.6	0.0	0.9	5.4	0.0
BRS	0.1	0.0	0.0	0.1	0.1	0.0	0.2	0.0	0.0	0.1	0.0	0.0
Wb	0.2	0.2	0.0	0.2	0.2	0.0	0.0	0.1	0.0	0.0	0.0	0.0
Wl	0.4	0.1	0.0	0.0	0.1	0.0	1.9	1.4	0.1	0.3	1.5	0.1
CLS	1.9	4.0	0.7	1.6	6.3	0.5	0.1	2.6	0.6	0.2	3.0	0.3
FP	2.7	0.4	0.0	1.1	0.5	0.0	1.8	2.1	0.0	1.5	3.0	0.0
UrBu	4.9	0.8	0.0	4.9	2.0	0.0	0.0	0.2	0.1	0.0	0.2	0.0
Overall	7.7	10.3	3.3	6.7	14.4	2.4	6.3	10.1	1.8	1.7	12.4	1.4

Even though applied to different land cover maps than proposed (Pontius and Millones, 2011; Pontius and Chen, 2006), the disagreement budget provides a comparison between the measured land cover maps and the future scenario for which there is no validation data.

**Fig. 5:**
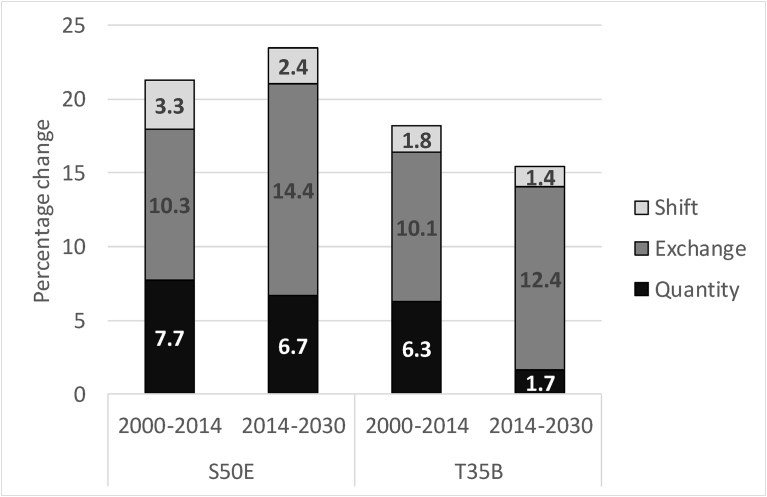
Disagreement budget.

Table 10 reveals the classes that account for the largest exchanges and therefore possibly the largest model errors. In both measured and modelled transitions, UG had the highest exchange percentage approximately 9% for the 2000–2014 transition and ∼12% for the modelled transition (in bold italics). In the measured data (2000–2014) the similarity between categories with similar spectral signatures could cause exchange error, which would be propagated to the predicted LULCC model. Fig. 5 shows the overall disagreement budget of the catchment at the two time steps for the two catchments.

Fig. 5 shows the increase in exchanged pixels in the predicted LULCC model for 2030, with lower quantity disagreement, particularly in T35B. The similarity in quantity disagreement between measured and modelled scenarios implies that for S50E the correct number of pixels were allocated to a class. The high exchange disagreement for classes UG, FITBs and CLs, as well as FP in T35B suggests that these classes may not be accurately modelled in the 2030 land cover map and that certain transitions were incorrectly predicted. This may be expected based on the model accuracies reported for S50E in Table 4 with none of the sub-models having an accuracy level higher than 70%. For T35B (Table 5), sub-models for urban intensification (Iu) and afforestation (R) provided accuracies of higher than 80%. The disagreement budget for these classes in the two time periods is also similar. The sub-models for abandonment (A) and natural dynamics (Dn) presented accuracies lower than 50%, but very few pixels were associated with these transitions. Despite these shortcomings, the overall proportion of the land cover within the catchment is likely to be reasonably accurate.

Aldwaik and Pontius (2012) note that when a large dominant class exists, accounting for a large percentage of the study area, other classes may appear more active by comparison. However in this study area, the largest class UG is involved in substantial modelled change, and cannot be excluded. In S50E, active transitions occurred from FP (D), FITBs (Re) and CLs (A) to UG with a hypothesized error of 3%, while UG was the target of intensification to FITBs (If) and UrBu (Iu). The intensification of FITBs is regarded as a systematically targeting transition as a gain in FITBs targets UG while FITBs also targets the loss of UG. The same holds true for urban intensification (Iu). This interchange of classes may contribute to the high exchange disagreement. In T35B, FITBs intensification (If) and reclamation (Re) systematically targeted UG, implying an exchange of FITBs and UG over the prediction period.

## 6 Discussion

Land cover change, which is closely linked to rural development initiatives, presents challenges for integrated land and water resources management in the Eastern Cape. The aim of this research was to project land cover change trends into the future (2030) to gain an understanding of the implications on biophysical parameters which in turn can guide land management strategies. However the complex processes of land cover change are difficult to capture in variables, and model in algorithms, since they are often shaped by dynamic, non-linear human-nature interactions (Camacho Olmedo et al., 2015). For this reason, the discussion will firstly focus on potential sources of error in the LCM and then, with these potential limitations in mind, the focus shifts to the implications of the land cover trend projection on biophysical parameters, should no interventions be implemented.

A land change model must predict both the quantity of each land cover type as well as the location of any change (Pontius et al., 2004). The accuracy of an inductive model's output is a function of both the model itself i.e. suitability of algorithms within the model to fulfil the intended purpose, and the accuracy of the input data. Thus to anticipate where possible inaccuracies may be entering into modelled output, assumptions within the model can be examined, as can accuracies of input data. Fig. 2 shows a flow diagram of the approach taken in LCM which is useful to view in light of this discussion.

Since the LCM is an inductive approach, past land cover maps are used to empirically model change. Errors in the individual input land cover maps will be propagated through the model and produce errors in both future scenario output and the potential to transition map. This study is a follow on from the change analysis carried out by the same authors where the land cover maps, their accuracies, and the implications of these accuracies in change analysis are described (Münch et al., 2017). The overall accuracies for the land cover maps was reported as 83 and 87% for S50E and 81 and 83% for T35B for T_1_ and T_2_ respectively and these levels of accuracy equate to a change accuracy of 67% for T35B and 72% for S50E. This may appear rather low however if higher change accuracies are required, for example, change mapped with 75% reliability, the accuracy of input land cover maps at T_1_ and T_2_ would need to be about 90% (Fuller et al., 2003), a seldom achievable accuracy level when using mapping land cover from medium resolution satellite imagery. Suffice to say, land cover classification is fraught with uncertainties (Münch et al., 2017) and these uncertainties are propagated through to errors in historic change quantification and indeed future scenario mapping too. It is therefore important to take cognisance of this limitation and any interpretation of results should be with these accuracies in mind.

Within the LCM, past land cover spatial distribution is used to estimate both potential to transition and a future land cover scenario as a function of explanatory spatial variables through mathematical modelling (Mas et al., 2014). This modelling is based on two assumptions. Firstly, in the Markov projection, rates of change are assumed to be constant implying that external forces exerting the change remain constant too. In context of increasing human pressure on the land, climate change, and variability in rainfall *inter alia,* this assumption can be flawed. Over short time periods, the impact of the rate of change may not have a significant impact on the projected change, as described by Roy et al. (2014), especially when the scale of the input land cover maps is considered. However, climate patterns may be cyclical and if change maps T_1_ and T_2_ represent different stages within that cycle (e.g. one in a particularly wet year and the other in a particularly dry year) then rate of change may show much higher than may otherwise be predicted, with the inverse also being true.

Secondly, in LCM the drivers of change (explanatory spatial variables) are assumed to act identically to create the propensity for change maps. Cramer's V is used to test the level of association between a potential explanatory spatial variable and the historic change recorded between T_1_ and T_2_. The user then decides based on his/her own expert knowledge, the explanatory variables which should be allocated to each transition sub model using Cramer's V as a guide to which variables to include, which in turn will impact on the resulting change propensity map. Then even though some explanatory spatial variables may better describe the historic change, once the variable is selected into a sub model, the model does not rank or weight the variable on the basis of its usefulness in describing past change. Thus should a variable with a low Cramer's V be selected, it will have as much influence on the change propensity map as a variable with a high Cramer's V. Much research in land change modelling has been based on comparing the outputs between different models (e.g. Mas et al., 2014) however, if a single model can produce different outputs based on the users choice of model parameters, there can be greater variation within the outputs of a single model than between the outputs of different models (Camacho Olmedo et al., 2015). Furthermore, the spatial explanatory variables are implemented in the model as stable over time, thus they will have the same influence at T_2_ all the way through to T_3_. In reality this is unlikely to be the case as some of these variables may also change over time, a topic explored in more detail by Kolb et al. (2013).

Despite these limitations, in the context of trying to understand appropriate land management interventions for both catchments in light of the trends presented in the results, a qualitative discussion of biophysical parameters that impact on catchment water use, NEE and the surface energy balance and the expected land cover transitions is presented. Firstly, from a water resource management perspective, globally, >66% of the total precipitation over land is returned to the atmosphere as ET (Fisher et al., 2005; Mu et al., 2011; Hoff et al., 2010; McMahon et al., 2013; Liou and Kar, 2014) which makes ET very important in catchment water balance. During photosynthesis, plants accumulate new biomass as they release water in exchange for atmospheric carbon and ET rates are closely related to the carbon assimilation rates of plants (Franks et al., 2013). It is well established that knowledge of land cover can give insight (via ecosystem surface conductance and ET) into the water use of the land surface. A transition towards land covers with higher surface conductance will result in higher water use via ET in the catchment. In this study the loss of grasslands favouring an increase in anthropogenic land covers (agricultural and FITBs intensification) will result in higher catchment ET for both S50E and T35B with T35B being most impacted.

Next, from a carbon perspective, fPAR and LAI measured by Palmer et al. (2017) and used in NEE and ET modelling respectively, indicate that both fPAR and LAI are lower for un-improved grasslands than for potential transition classes (Intensification of FITBs and Intensification of CLS and afforestation). These transitions will thus represent a gain in both catchment NEE and ET, and a concomitant decrease in run-off. From a carbon storage perspective, the transitions will result in more carbon storage, which from a climate change outlook may be seen as a positive change, however in an already water scarce catchment, further water demands by the vegetation will result in a decrease in the availability of water for other land covers.

Finally when considering the surface energy balance, the changes to surface albedo that will accompany these land cover trajectories are less certain. Given that there is a general increase in woody green biomass as a result of both afforestation and continued invasion by IAPs, the findings of Rotenberg and Yakir (2010) make it likely that the decrease in surface albedo from these cover classes will result in an increase in the absorption of energy, with a resultant rise in temperature. This decrease in albedo may however be counteracted by an increase in degraded surfaces associated with rural housing and in the unimproved grasslands where continuous grazing by livestock changes species composition and cover. Rangeland degradation is commonly associated with the changes in land tenure that are occurring in this catchment (Bennett et al., 2012), and a reduction in the basal cover of herbaceous plants (mainly grasses) is the first noticeable change. This results in a surface with higher albedo.

Since, the land surface reflectance (albedo) affects net surface radiation, dark vegetation with a high LAI will have a lower albedo than open grasslands and it can be postulated (however this must still be measured) that the transitions modelled in the S50E catchment could lead to an overall lowering of albedo in the catchment. An increase in net radiation is thought by some to be a driver of global warming, however, Bonan (2008) states that surface warming arising from the low albedo of forests is offset by strong evaporative cooling. Thus the impact of a change in albedo in this catchment remains speculative but is a research avenue which should be pursued.

## 7 Conclusion

In this paper, the Land Change Modeller (Idrisi Terrset 18.08) was used, together with land cover mapped for the years 2000 and 2014 (Münch et al., 2017), to model land cover for the grassland dominated S50E and T35B catchments in the Eastern Cape Province for the year 2030. It has been postulated that future land cover changes may result in adjustments to biophysical drivers impacting on NEE and catchment water use through ET. This work has thus built on previous work (Palmer et al., 2017; Münch et al., 2017; Gwate et al., 2016; Okoye, 2016) as a first step in determining the impact of future land cover change on catchment water and carbon fluxes.

It was found that in 2014 for **S50E** *(T35B)*, grasslands represented **57%** *(80%)* of the total catchment area with this figure modeled to decrease to **52%** (*80*%) by 2030 with losses likely to favour a gain in woody plants and cultivated land. The results show that the total change (gain and loss) in the landscape over all land cover classes was **21%** *(18%)* for the period between 2000 and 2014 and **23%** *(16%)* from 2014 up to the future scenario for 2030, with the change intensity remaining constant at **1.5%** *(<1%)* per year. It was determined that the probability of grasslands persisting is around **80%** *(>90%)* with the highest probability of grasslands being lost to woody encroachment **∼5%** *(3%)* and cultivation **∼7%** *(<2%)*.

Since fPAR and LAI are lower for grasslands than for their transition classes (Palmer et al., 2017), these transitions represent a gain in both catchment NEE and ET, resulting in increased carbon storage, and corresponding increased water use by vegetation. It is postulated that these carbon offsets from sequestration may be counterbalanced by temperature increases linked to lower albedo increasing net surface radiation and it is this carbon-water-surface energy flux nexus that requires further research in quantifying impacts. The higher LAI will undoubtedly increase catchment-scale ET and reduce run-off. The lower albedos will increase surface temperature, and although these may be offset by higher albedo from urbanized and degraded surfaces, the net result from an increase in woody biomass will be a catchment with a lower capacity to provide water to its residents or downstream users.

The LCM models future scenarios based on trends of historic change and therefore the results represent a future scenario based on no intervention deviating from past interventions. The impact of the different land management practices in S50E (dualistic farming system) and T35B (commercial system) can be identified in the historic land cover change trends and in the future scenario. It is apparent that under the dualistic farming system, degradation is taking place at a more rapid rate than in T35B where over 90% of current grassland is expected to persist to 2030. For those involved in planning in these rural catchments, there should be greater sensitivity amongst policy makers towards the negative effects of further afforestation and uncontrolled invasion of IAPs. Finally, the results suggest that rehabilitation and land management initiatives should be targeted in catchments under a dualistic farming system, rather than those which are predominantly commercial systems.

## Declarations

### Author contribution statement

Lesley Gibson, Zahn Münch: Conceived and designed the experiments; Performed the experiments; Analyzed and interpreted the data; Contributed reagents, materials, analysis tools or data; Wrote the paper.

Anthony Palmer, Sukhmani Mantel: Conceived and designed the experiments; Wrote the paper.

### Funding statement

This work was supported the Water Research Commission [Grant no: K5/2400/4/3].

### Competing interest statement

The authors declare no conflict of interest.

### Additional information

No additional information is available for this paper.
